# The changing landscape of urothelial carcinoma: on the edge of a paradigm shift

**DOI:** 10.1172/JCI202079

**Published:** 2026-03-02

**Authors:** Joshua J. Meeks

**Affiliations:** 1 Departments of Urology and; 2 Biochemistry and Molecular Genetics, Northwestern University, Feinberg School of Medicine, Chicago, Illinois, USA.; 3 Jesse Brown VA Medical Center, Department of Veterans Affairs, Chicago, Illinois, USA.

## Abstract

Urothelial cancers of the urinary tract are the fourth most common malignancy in men, with a shifting demographic affecting younger patients and an increasing incidence in females. In this Review, we discuss recent discoveries and paradigm-shifting clinical trials that impact all stages of urothelial cancer. New therapeutics and drug-delivery devices have led to multiple approvals for treatments of non-muscle invasive bladder cancer. The addition of chemotherapy, immunotherapy, and antibody-drug conjugates is transforming perioperative treatment for patients with muscle-invasive bladder cancer. The use of liquid biomarkers, circulating tumor DNA, and urinary tumor DNA is aiding the identification of patients at risk for local recurrence and possibly those who can avoid systemic therapy. Finally, integrating biomarkers and systemic treatments is creating a paradigm that could lead to the successful treatment of bladder cancer without requiring bladder removal. Overall, these advancements in biomarkers and novel therapeutics are likely to dramatically improve survival for bladder cancer.

## Introduction

Urothelial carcinoma comprises epithelial tumors of the urinary tract: the renal pelvis and ureter (15% of cases; upper urinary tract), the bladder (80% of cases; lower urinary tract), and the urethra (5%, including the prostatic urethra to the meatus) (1). Bladder cancer is the fourth most common cancer in men in the United States and is the tenth most common worldwide across sexes (2, 3). In the United States alone, over 84,000 new cases are diagnosed each year, and more than 744,000 people are living with bladder cancer (4). Although bladder cancer has historically occurred with a 3:1 male predominance, we are observing a demographic shift: a 1% annual decrease in male incidence in many parts of Europe, along with a significant trend toward earlier diagnosis in patients under 50 years old (hazard ratio [HR] 1.34, 95% CI 0.6–2.07, *P =* 0.003) (2). Bladder cancer was once primarily considered a smoking-related cancer; however, unlike lung cancer, only 50% of bladder cancer patients have a history of smoking, and the direct correlation between pack-years and mutation rate is nonlinear (5). Moreover, while the incidence and mortality rates of lung cancer have decreased due to smoking cessation and screening, comparable improvements have not been observed in bladder cancer (4). The persistent incidence and steady mortality suggest that the mechanisms of carcinogenesis in bladder cancer may differ from those in lung cancer and are likely independent of smoking (6). In the absence of a clear etiology and effective strategies to screen high-risk yet asymptomatic patients, future advances in bladder cancer are likely to depend on new therapeutics and biomarkers for patient selection (7). This Review emphasizes innovative paradigms, emerging technologies, and targeted treatments that are rapidly transforming the landscape of bladder cancer.

## Early-stage cancer: novel therapies

Almost 75% of bladder cancers are “superficial” tumors corresponding to the stages noninvasive papillary carcinoma (Ta), tumor invading connective tissue (T1), and carcinoma in situ (Tis). Unlike tumors that invade the deeper layers of the bladder (T2), the risk of progression to metastasis with superficial, or non–muscle-invasive bladder cancer (NMIBC) is low (5%–10% at three years) (1). Yet, NMIBC recurrences are frequent and difficult to prevent, with 60% of NMIBCs recurring within three years of diagnosis (8). The high risk of recurrence of NMIBC is secondary to the unseen, multifocal, and often “pan-urothelial” extent of cancer-specific mutations (e.g. *KMT2D*, *TP53*, and *TERT*) that have been identified in normal cells throughout the bladder by deep sequencing (9, 10). While field cancerization has been described in urothelial cancer and is associated with NOTCH2 mutations, it is not clear whether mutations in *TERT* or *TP53* occur in bladders that do not develop cancer (11). The early mutational changes are hypothesized to result in a histologically flat lesion described as carcinoma in situ (CIS, or stage Tis). Thus, topical therapies that treat the entire urothelium have become a cornerstone of treatment (12).

**Figure 1:**
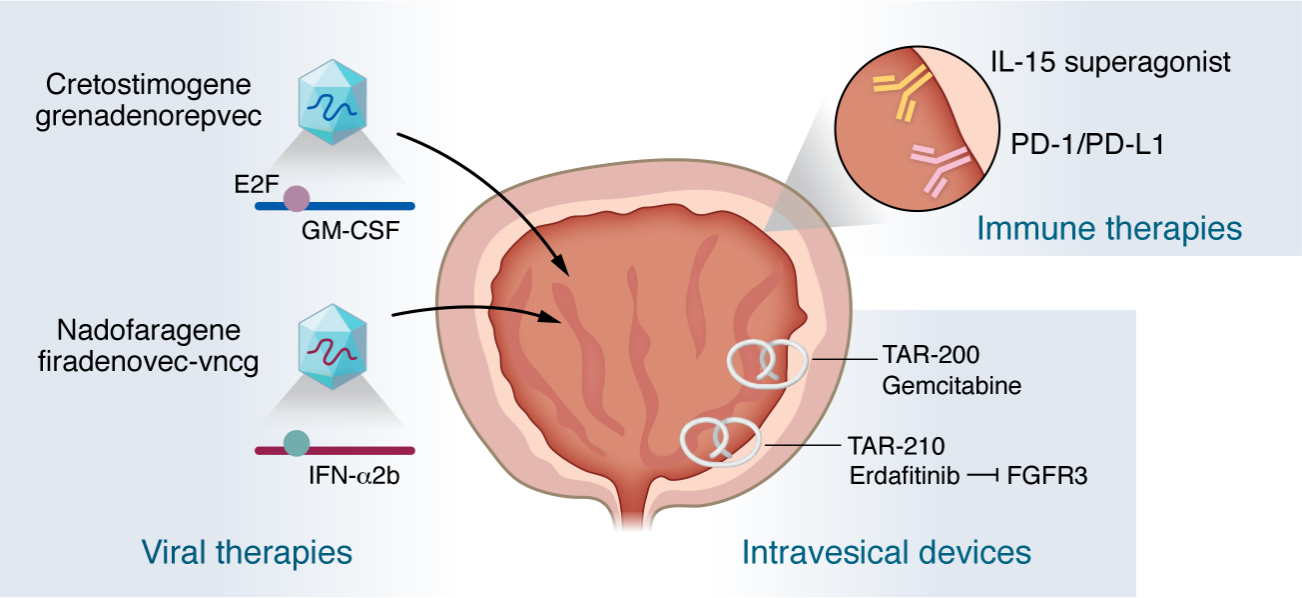
Novel therapies for early-stage bladder cancer. Multiple new therapeutic strategies are in development for the treatment of non–muscle-invasive bladder cancer. To initiate anticancer immune responses, viral therapies have been developed to drive expression of cytokines, including IFN-α and GM-CSF. Coiled bi-oval devices have been designed to enable sustained, local delivery of chemotherapy. In addition, immunotherapies, including checkpoint inhibitors, are being explored for their potential in bladder cancer, delivered either systemically or locally.

The oldest and most widely used therapy in NMIBC is the tuberculosis vaccine, Bacillus Calmette-Guérin (BCG) (13). Discovered in 1921 and first applied to bladder cancer in 1974, repeated administration of BCG develops trained immunity to direct both innate and adaptive immune responses to the bladder cancer, ultimately decreasing the frequency of both recurrence and progression (14). The immunologic response to BCG is driven by myelopoiesis in the bone marrow and depends on adaptive T cell immunity from CD4^+^ and CD8^+^ T cells (15). Interestingly, the T cell response to BCG is both tuberculosis and cancer specific, suggesting that BCG initiates immune activation but the overall response includes tumor-specific neoantigens. Between 25% and 40% of patients with NMIBC will not tolerate BCG or will experience recurrence, and until very recently, bladder removal was the only effective secondary treatment after BCG (16). In contrast to responders, the T cells of nonresponders express exhaustion markers, such as PD-L1 (17). Thus, several therapies have been developed to initiate a greater immunologic response by increasing cytokine production or targeting immune exhaustion (Figure 1). The first therapy that achieved a meaningful benefit was nadoferigene feridenovic (Adstiladrin) (18). Adstiladrin utilizes a recombinant adenoviral vector driving expression of IFN-α, with Syn3 excipient, and has documented activity for three months after instillation. In the single-arm phase III trial of 103 patients with BCG-unresponsive NMIBC that led to FDA approval in 2022, Adstiladrin achieved a 53% complete response rate, with 46% of patients remaining recurrence free at 12 months (19). Similarly, cretostimogene grenadenorepvec (formerly CG007) is an adenoviral vector with the E2F-1 promoter driving expression of the cytokine GM-CSF (20). The concept of cretostimogene therapy is to selectively drive expression of GM-CSF in cancer cells deficient in retinoblastoma (RB) protein with limited activity in wild-type urothelial cells. In the single-arm phase III BOND-003 trial in 112 enrolled (110 evaluated) BCG-unresponsive patients with NMIBC, cretostimogene achieved a complete response rate of 75.5%, and more than 46.4% of patients maintained in complete remission at 12 months (21). Collectively, the use of adenoviral factors provides a mechanism of sustained gene delivery (22). While viral gene therapies have faced challenges in other disease states, they have been readily adopted for NMIBC, with little concern about viral toxicity, likely due to their limited systemic exposure.

While single-agent chemotherapy achieved only a limited clinical response in historical trials, with a durable response of 18% at 12 months (23), a sustained delivery system in the form of a coiled bi-oval (pretzel) was developed for gemcitabine (24). This TAR-200 device (now called Inlexzo) allows the bi-oval to be unfolded into a wire, then placed into the bladder, where it recoils into the bi-oval form and is retained (25). The TAR-200 device provides sustained delivery over three weeks, but is loaded with gemcitabine at one-tenth the normal chemotherapy dose. In the single-arm SunRISe-I phase IIb trial in BCG-unresponsive CIS with or without papillary tumor, TAR-200 achieved an 82% response rate, with 50% of patients remaining in remission for 12 months (26). SunRISe-1 was a parallel-cohort study that compared TAR-200 with the PD-1 inhibitor cetrelimab (cohort 1) to TAR-200 monotherapy (cohort 2) or cetrelimab monotherapy (cohort 3). While cohorts 1 and 2 were effective, TAR-200 monotherapy (cohort 2) showed improved tolerability and fewer device removals due to side effects.

While simple in appearance, sustained delivery of an indwelling chemotherapy agent is a remarkable technological advance. A second-generation bi-oval delivery system is the erdafitinib-eluting device, TAR-210 (27). Erdafitinib targets FGF receptors; *FGFR3* is mutated in 11% of metastatic bladder tumors, but 70% of NMIBCs. As an oral therapy, erdafitinib has moderate efficacy, with only a 45% response rate, but therapy is often stopped because of off-target toxicity (28). In contrast, when erdafitinib is loaded into the bi-oval device, TAR-210 can achieve a 1,000-fold higher dose in the bladder, with almost no detectable serum levels (27). In a single-arm phase I/II trial, TAR-210 achieved an 82% response rate in patients with intermediate-risk NMIBC. Current translational research priorities include identifying mechanisms of resistance and exploring the potential of combination therapies.

Antibodies targeting immune checkpoints (check point inhibitors, CPIs) have revolutionized therapeutic strategies across all stages of bladder cancer and have recently been approved by the FDA for NMIBC (1). In the KN57 trial of 96 patients, treatment of BCG-unresponsive NMIBC with the CPI pembrolizumab alone in resulted in a complete response rate of 40%, with 46% still in remission at 12 months, and ultimately a 19% 12-month response (29). While there were no new safety signals in KN57, the finding of 13% grade 3/4 treatment–related adverse events was high for this population of patients with early-stage cancer. The addition of CPI to BCG-naive patients has consistently resulted in a 25%–35% improvement in recurrence-free survival across multiple clinical trials (30, 31). While these trials have not demonstrated improvement in progression-free or overall survival (OS), the use of CPI for at least 12 months alleviates some of the exhaustion associated with BCG, improving its durability. While no CPIs are currently approved for use with BCG, these positive phase III trials demonstrate the efficacy of CPI in early-stage NMBIC. We conducted a small, phase I trial instilling pembrolizumab into the bladder to avoid the toxicity of systemic exposure (32). To our surprise, we identified a fatal autoimmune disease, including one death due to myasthenia gravis. These anecdotal data suggest that the mechanism and side effects of CPI still require further investigation in early-stage bladder cancer.

## Perioperative systemic therapy

From diagnosis, the two-year survival rate for patients with locally advanced stage II–IVa or MIBC is only 50% (33). The recent implementation of systemic therapy has improved survival. Chemotherapy was first tested after surgery to determine whether an adjuvant strategy could decrease the risk of recurrence (34). Unfortunately, multiple trials of adjuvant chemotherapy closed before completion, secondary to the challenges in accrual after radical cystectomy (35). In retrospect, this was likely secondary to the physical burdens of the operation and the rapid progression of high-risk MIBC. To mitigate the challenges associated with recovery from cystectomy, a neoadjuvant approach, or systemic therapy prior to cystectomy, was evaluated. In a landmark trial comparing neoadjuvant chemotherapy followed by surgery to surgery alone, patients in the cystectomy-alone arm had a 33% greater risk of death than the neoadjuvant chemotherapy followed by surgery arm (HR 1.33, 95% CI 1.00–1.76) (36).

Since then, recent advances in perioperative therapy have included the addition of immunotherapy after surgery (1). Three large, randomized trials evaluated the benefit of 12 months of immunotherapy compared to placebo or observation (CM274, IMvigor010, and AMBASSADOR). The trial with the largest effect size was CM274, in which 709 patients were randomized to placebo or adjuvant nivolumab, which found improvement in disease-free survival (HR 0.74, 95% CI 0.61–0.90), but no significant improvement in OS (75.0 vs. 50.1 months; HR 0.83, 95% CI 0.67–1.02) (37). While the AMBASSADOR and CM274 trials found a decreased risk of recurrence, no trials have identified a survival benefit in unselected patients. In addition, although the IMvigor010 trial demonstrated no significant improvement in recurrence-free survival or OS for patients treated with 12 months of adjuvant atezolizumab, it did show a reduction in circulating tumor DNA (ctDNA) at treatment cycles 1 and 3 (discussed below) (38, 39).

The improvement in clinical response to adjuvant immunotherapy provided the first evidence that perioperative immunotherapy could improve survival for patients with MIBC, potentially by increasing the durability of response to chemotherapy by immune checkpoint blockade (40). The largest clinical trial of perioperative immunotherapy was the Niagara trial (41). In Niagara, over 1,000 patients were randomized to cisplatin-based chemotherapy (gemcitabine and cisplatin) compared to cisplatin-based chemotherapy and the PD-L1 inhibitor durvalumab. After cystectomy, patients on the durvalumab arm continued to receive durvalumab for an additional eight cycles. Niagara demonstrated that the addition of durvalumab, both before and after surgery, improved recurrence-free survival and OS, with an HR of 0.68 (EFS: 95% CI 0.056–0.82, *P <* 0.001; OS 0.75, 95% CI 0.59 to 0.93, *P =* 0.01 by stratified log-rank test). There was no greater toxicity in patients treated with durvalumab, and the rate of grade 3 treatment–related adverse events was the same in both arms (41% in each). The median time from completing chemotherapy to surgery was not affected by durvalumab (5.6 vs. 5.4 weeks), and the addition of immunotherapy did not increase complications at radical cystectomy. A biomarker analysis of Niagara found that more patients achieved ctDNA clearance with durvalumab and chemotherapy than with chemotherapy alone (70% vs. 57%) (42). ctDNA clearance was associated with greater pathologic response (51% pathological complete response [pCR] in ctDNA-negative patients vs. only 3% pCR in ctDNA-positive patients) and baseline ctDNA positivity was prognostic (EFS HR 0.42, 95% CI 0.30–0.60). The Niagara study resulted in greater use of chemotherapy and immunotherapy for MIBC. Yet, the trial that will likely redefine the approach to MIBC is the ongoing KN905/EV303 trial (ClinicalTrials.gov NCT03924895), comparing the antibody-drug conjugate (ADC) enfortumab vedotin (EV) with pembrolizumab alone or with cystectomy in patients unable to receive chemotherapy.

## ADCs revolutionize cancer therapy

**Figure 2:**
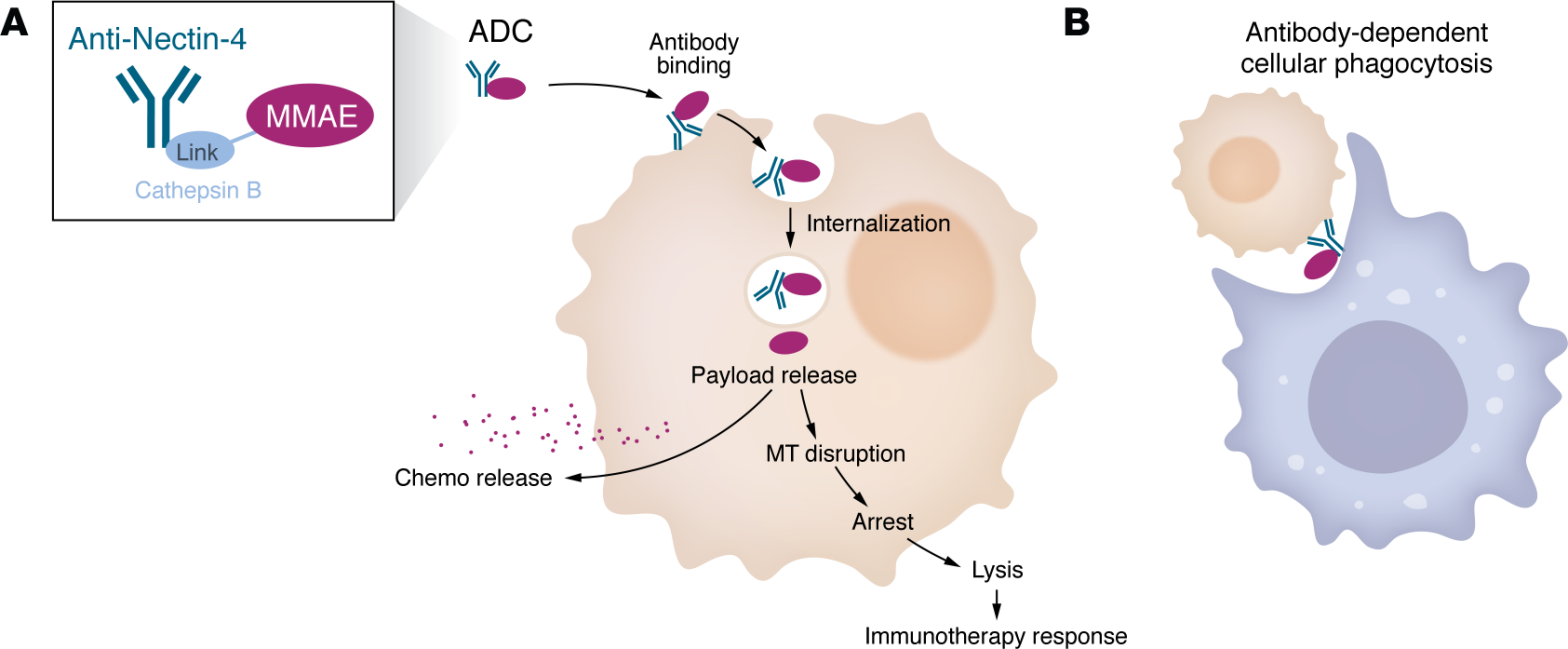
Antibody-drug conjugates. (**A**) Antibody-drug conjugates (ADCs) couple antibodies targeting cancer cells to cytotoxic payloads. The ADC enfortumab vedotin comprises an anti–NECTIN-4 antibody attached to a linker carrying the MMAE payload, which disrupts microtubules (MTs). (**B**) Binding of ADCs to cancer cells can also enable antibody-dependent phagocytosis by macrophages.

EV was the first ADC to demonstrate therapeutic activity in bladder cancer (43). EV targets NECTIN-4, which is widely expressed in the urothelium, and is often expressed at sites of metastatic urothelial cancer (44). The anti–NECTIN-4 antibody is attached to a linker carrying the monomethyl auristatin E (MMAE) payload, which disrupts microtubules (Figure 2). EV demonstrated a survival advantage compared with other second-line treatments (median OS 12.88 vs. 8.97 months, HR for death 0.70, 95% CI 0.56–0.89, *P =* 0.001) when used as a single agent after progression from chemotherapy and immunotherapy (45). The landmark EV302 trial compared the addition of pembrolizumab to EV (EVP) with systemic chemotherapy in patients with new metastasis (1L) or locally advanced cancer (46). EVP provided more than a two-fold improvement in survival (median 31.5 months vs. 16.1 months, HR for death 0.47; 95% CI 0.38–0.58, *P <* 0.001). The FDA approved EVP for 1L bladder cancer in 2023. With a response rate approaching 65% and no effective alternative therapy, there has been limited biomarker research to identify patients more or less likely to respond to EVP. NECTIN-4 expression is complex, with nuclear, cytoplasmic, and membranous localization described in urothelial cancers (47). Membranous NECTIN-4 is associated with a greater response in EV-treated patients, with other cellular locations associated with resistance (moderate to strong expression, HR 4.26, 95% CI 1.55–11.70, *P* = 0.005) (47). Further evaluation of *NECTIN-4* amplification, which is found in 18% of MIBC and 26% of metastatic urothelial cancers, is associated with increased NECTIN-4 expression and a greater response to EV with an HR of 0.08 (95% CI 0.02–0.34) (48). Building on the activity of EVP in metastatic urothelial carcinoma, two trials of perioperative EVP (for stage II–IVa MIBC) evaluated the clinical response of EVP compared to surgery alone (EV303) or cisplatin-based chemotherapy (EV304). Compared with surgery, EVP had a pCR rate of 57%, and significantly improved EFS (HR 0.40, 95% CI 0.28–0.57, one-sided *P <* 0.001) and OS (HR 0.5, 95% CI 0.33–0.74, one-sided *P =* 0.002) (49). The results of EV303 changed the perioperative paradigm for MIBC. First, EVP was the first non–cisplatin-based chemotherapy regimen with activity in MIBC, removing the cisplatin requirement for neoadjuvant therapy. Thus, more patients now qualify as candidates for perioperative therapy. Second, the increased pathologic response with EVP now presents the possibility of omitting surgery in complete responders.

Another ADC family targets the human epidermal growth factor receptor 2 (HER2) (50). HER2 is overexpressed in 78% of bladder cancers, and there is a correlation between luminal differentiation and HER2 expression (51). While the original HER2 ADC, trastuzumab emtansine, showed limited response in urothelial carcinoma (52), newer HER2-targeting ADCs such as disitamab vedotin used in combination with toripalimab had a response rate of 76% in 1L cancers, with greater progression-free survival (median 13.1 vs. 6.5 months, HR 0.36, 95% CI 0.28–0.46, *P <* 0.001) in HER2+ (IHC 1+, 2+ or 3+) compared with chemotherapy (53).

## Chasing gold: liquid biomarkers

**Figure 3:**
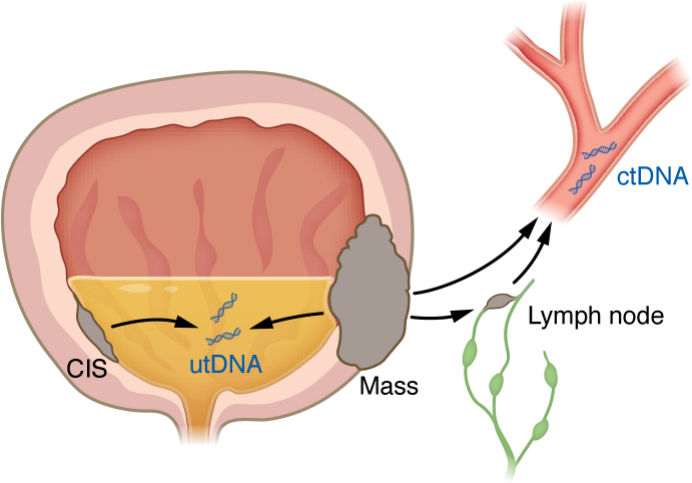
Liquid biopsies. Advances in the ability to detect and analyze urinary tumor DNA (utDNA) and circulating tumor DNA (ctDNA) now enable stratification of patients into high and low risk for recurrence and interrogation of local and systemic recurrence. ctDNA is more specific, and usually a sign of advanced cancer, whereas utDNA is more sensitive, with a high negative predictive value.

Despite close access to the bladder and ample production of urine, a major challenge for bladder cancer has been the development of accurate liquid biomarkers (Figure 3). Over time, advances in molecular techniques have led to the description of three distinct generations of urine biomarkers (54). The first generation of urinary biomarkers were specific protein(s) present in bladder cancer compared with controls. While specific for bladder cancer, these biomarkers often lacked sensitivity to detect early cancers and low-volume tumors. Second-generation biomarkers were identified via molecular analysis of 10 or more cancer-enriched genes, offering greater sensitivity but still lacking accuracy in heterogeneous samples. Third-generation urine biomarkers are based on evaluation of the entire genome or targeted exome. These next generation sequencing–based assays include a gene panel, RNA-seq–based evaluation of copy number variation, and low-pass whole-genome sequencing. This technology enables sufficient sensitivity to identify individual gene copy numbers in urinary tumor DNA (utDNA).

Most urine-based molecular testing can be categorized into two functions. First, at the initiation of intravesical treatment after bladder tumor resection, urinary biomarkers can be used for risk stratification, placing patients into high- or low-risk groups based on the presence or absence of a defined number of mutations or detectable utDNA levels. In this case, the marker is prognostic (55). A second function is to evaluate on-treatment dynamics to determine whether the therapy results in improved utDNA clearance, identifying patients at risk for recurrence. This would allow intervention weeks to months prior to a more standard approach. An example is the UroAMP biomarker, developed by Convergent Genomics, which includes sequencing of 60 bladder cancer–specific genes and low-pass whole-genome sequencing (56). UroAMP is not tumor-informed. Samples are classified as “high” or “low” risk based on a previously developed algorithm, and the summation of mutations, described as genomic disease burden (GDB), is calculated, which correlates to the number of mutations found in the sample including copy number, aneuploidy, single-nucleotide variants, and indels. UroAMP has been evaluated in multiple retrospective cohorts (57), including two clinical trials in BCG-unresponsive NMIBC, one with atezolizumab (S1605) (58) and the other with Adstiladrin (59). In both cases, the minimal residual disease (MRD) status before treatment ranged from 69% to 72% with detectable utDNA. The strength of the UroAMP assay is its bladder-specific gene set, which is agnostic to patient mutations and eliminates the need to develop a patient-specific gene panel. In contrast, deeper sequencing has been reported for urine assays from Predicine and Natera, potentially increasing sensitivity. For example, Predicine developed BEACON based on whole-exome sequencing (WES) of the tumor or urine, boosting coverage of 600 cancer genes from the baseline sample, to design a personalized MRD panel tracking up to 50 mutations with a fixed panel of 500 actionable mutations (60). Using tissue-based WES, Natera developed a urine assay for utDNA. The benefit of the Natera assay is that it can use the tissue-based WES for the ctDNA assay to provide an orthogonal evaluation of both ctDNA and utDNA, enabling interrogation of systemic and local recurrence.

A major clinical advance in bladder cancer was the identification of ctDNA shed into the blood from either the bladder or metastatic sites. ctDNA detection correlates with stage, as 50%–60% of patients with MIBC are ctDNA-positive, compared with only 35% of stage I patients (61) by Signatera. In patients who were ctDNA-positive after bladder removal, the overall and 12-month recurrence rates were 76% and 59%, respectively, whereas in ctDNA-negative patients, the rate was 0% (62). The first wide-scale evaluation of ctDNA in a clinical trial was IMvigor010, an adjuvant trial of atezolizumab after radical cystectomy. IMvigor010 included 581 patients with ctDNA drawn at the start of treatment cycles 1 and 3 (abbreviated C1D1 and C3D1) (39). After cystectomy, Signatera ctDNA at C1D1 was found in 37% of patients and resulted in a significantly greater risk of recurrence (observation arm HR 6.3, 95% CI 4.45–8.92, *P* < 0.0001). Intriguingly, only ctDNA-positive patients had improved disease-free survival and OS with adjuvant atezolizumab (disease-free survival HR 0.58, 95% CI 0.43–0.79, *P* = 0.0024; OS HR 0.59, 95% CI 0.41–0.86). These data suggest that selective treatment of ctDNA-positive patients with adjuvant immunotherapy can both reduce therapy-related toxicity for patients with limited response and offer selective benefit for those at higher risk of recurrence. For those ctDNA-positive patients treated with atezolizumab, a greater decrease in C3D1 ctDNA was associated with longer survival (100% clearance, 60.0 months [95% CI 35.5–not estimable]; 50%–99% reduction, 34.3 months [95% CI 15.2–not estimable]; <50% reduction, 19.9 months [95% CI 16.4–32.2]). Finally, the combined ctDNA positivity of C3D1 and C1D1 showed greater sensitivity than C1D1 alone (68% vs. 57%) (63).

The first prospective trial with ctDNA-guided treatment was TOMBOLA, which enrolled patients who were ctDNA-negative after cystectomy with a greater risk of recurrence (64). Patients were followed with ctDNA measurements every 3 months until ctDNA was detectable. Upon detection, atezolizumab was initiated for one year. This treatment strategy at the time of ctDNA detection successfully converted 55% of patients to ctDNA negativity, with no metastases detected. The initiation of therapy was formally tested in a randomized trial, IMvigor011 (65). As a follow-up adjuvant trial that enrolled high-risk patients like IMvigor010, ctDNA was evaluated at enrollment in 760 patients. Of the 286 Signatera ctDNA-negative patients, 171 met the criteria (time of enrollment) for analysis. Only 15 events occurred in ctDNA-negative patients, with 92% disease-free survival at 12 months. In those patients who converted to ctDNA-positive and were randomized to atezolizumab, there was a statistically significant improvement in disease-free survival (HR 0.64) and OS (HR 0.59) compared with placebo. One of the most interesting and definitive trials testing the integral nature of ctDNA will be the MODERN study (ClinicalTrials.gov NCT05987241). In this trial of high-risk MIBC, patients will have Signaera ctDNA testing after cystectomy. The ctDNA-positive patients will be randomized to either adjuvant nivolumab or nivolumab and relatlimab to determine whether intensification of adjuvant immunotherapy improves recurrence-free survival. Alternatively, ctDNA-negative patients will be randomized to observation or nivolumab to determine whether adjuvant treatment is beneficial.

## Organ preservation

**Figure 4:**
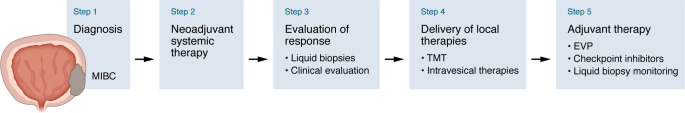
Framework for organ preservation. By combining multiple advances described in this Review, successful organ preservation will be possible in the near future. Step I is accurate staging of localized cancer using MRI, ctDNA, and utDNA. Step II is delivery of targeted and effective ADC systemic therapy combined with checkpoint blockade. Step III is the accurate evaluation of response by clinical evaluation and liquid biopsies. Step IV is the delivery of local therapies, which range from TMT to intravesical therapies (e.g. TAR-200). Finally, Step V is adjuvant therapy, which can include checkpoint and/or further ADC with liquid biopsy monitoring.

Until recently, due to the high risk of local recurrence, in the United States the primary local therapy for stage II–IVa bladder cancer was cystectomy. Outside the United States, bladder preservation was more common, with variable success rates, due to differences in patient selection, concomitant chemotherapy, and radiation therapy. An increased interest in bladder preservation has emerged in the United States, driven primarily by patient advocacy, improved radiation strategies, and a multidisciplinary approach to bladder cancer. Bladder preservation is often called “trimodal therapy” (TMT) for treatment that includes endoscopic resection, radiotherapy, and chemotherapy (1) (Figure 4). The primary endpoint for most bladder-sparing therapy is bladder-intact event-free survival (BI-EFS), which is a composite endpoint of invasive recurrence, metastatic cancer, surgical removal for poor bladder function or recurrence, and death from any cause. Unfortunately, a randomized trial of cystectomy compared to TMT was initiated but closed after only 45 patients were enrolled over 2.5 years (out of 1,015 target accrual), mainly due to patients’ refusal to be randomized and strong patient preference for TMT (66). The change in perspective on bladder-sparing coincided with the development of improved systemic therapies, namely immunotherapy, which was hypothesized to pair with TMT through the abscopal effect. A single-arm phase II trial of TMT and pembrolizumab demonstrated that hypofractionated radiotherapy with twice-weekly gemcitabine was well tolerated, with 6% (3 out of 54) gastrointestinal grade 3 or greater toxicity and only 12 (22%) recurrences (67). A second randomized trial sponsored by the Hellenic GU Cancer Group comparing nivolumab addition found improved BI-EFS (72% vs. 47%, *P =* 0.021), with no toxicity (68). In 2016, cooperative groups SWOG and NRG initiated the largest multi-institutional randomized trial of bladder preservation, SN1806 (ClinicalTrials.gov NCT03775265). SN1806 enrolled 475 patients and randomized patients to TMT versus TMT with atezolizumab. In a separate trial, a similar design was initiated, with the goal of randomizing 636 patients to TMT versus TMT with pembrolizumab for 2 years after TMT completion (ClinicalTrials.gov NCT04241185) (69).

The improved pathologic response following perioperative systemic therapies has challenged the concept that radical resection is critical to achieve survival for locally advanced bladder cancer and raised the hypothesis that bladder preservation is safe and feasible. One trial, S2427, was initiated in 2025 in patients with a measurable clinical response (cT1 or less) after neoadjuvant therapy in which patients were treated with TMT and pembrolizumab with an anticipated BI-EFS of 70% at three years (ClinicalTrials.gov NCT07061964).

While TMT is one bladder-preserving strategy, several investigators have begun to question whether local therapy is necessary for MIBC in patients who experience a complete response after neoadjuvant therapy. The first trial stratified patients by DNA damage repair changes and a complete response to neoadjuvant therapy. This single-arm trial was designed to evaluate whether the biomarker of DNA damage response could identify those likely to respond to systemic therapy (ClinicalTrials.gov NCT03609216) (70). RETAIN was a single-arm trial designed to determine whether bladder preservation could be achieved in patients with the same biomarker of DNA damage changes and a complete response to accelerated methotrexate, vinblastine, doxorubicin (Adriamycin), and cisplatin treatment. Of 78 patients, 25 were mutation-positive, and 8 out of 25 in active surveillance remained without surgical intervention, with a 62% metastasis-free survival rate (ClinicalTrials.gov NCT02710734) (71). An update of RETAIN (RETAIN II) added perioperative nivolumab and reported an early metastasis-free survival of 82% in the active surveillance cohort (72). In a biomarker-unselected cohort, 72 patients were prospectively enrolled to receive neoadjuvant GC-nivolumab for 4 cycles (73). A complete response was identified in 33 out of 72 patients. Of those that achieved a complete clinical response, only two of the 33 developed metastases at 24 months. These provocative data suggest that response to systemic therapy in the bladder is a potential biomarker for systemic response that can be used to determine bladder preservation.

## Future directions

The potential improvement in survival and recurrence for patients diagnosed with bladder cancer will likely drastically change how bladder cancer is managed in the very near future. Research that integrates potent but targeted treatments with biomarkers to select those most likely to benefit will shape the precision delivery of care. Next-generation trials that begin to implement these biomarkers in a risk-adapted fashion will have the greatest impact on those directly impacted by bladder cancer. All of the advances described will likely combine to improve the clinical outcomes for patients. For example, potent ADCs can decrease the lethality of MIBC, confirmed by liquid biopsy (ctDNA) ensuring localized cancer. This would then shift therapies to bladder-only treatments that include novel intravesical therapies for those patients with MRD detected by utDNA.

## Funding support

This work is the result of NIH funding, in whole or in part, and is subject to the NIH Public Access Policy. Through acceptance of this federal funding, the NIH has been given a right to make the work publicly available in PubMed Central.

- Department of Veteran Affairs grants BX005599 and BX003692.
- Department of Defense Impact Award no. HT94252410507.
- National Cancer Institute/NIH grant R01CA298333.

## Version 1 03/02/2026

Electronic publication

## References

[1] Flaig TW (2022). NCCN Guidelines® insights: bladder cancer, version 2.2022. J Natl Compr Canc Netw.

[2] van Hoogstraten LMC (2023). Global trends in the epidemiology of bladder cancer: challenges for public health and clinical practice. Nat Rev Clin Oncol.

[3] Jubber I (2023). Epidemiology of bladder cancer in 2023: a systematic review of risk factors. Eur Urol.

[4] Siegel RL (2025). Cancer statistics, 2025. CA Cancer J Clin.

[5] Freedman ND (2011). Association between smoking and risk of bladder cancer among men and women. JAMA.

[6] Fantini D (2019). Molecular footprints of muscle-invasive bladder cancer in smoking and nonsmoking patients. Urol Oncol.

[7] Lobo N (2022). Epidemiology, screening, and prevention of bladder cancer. Eur Urol Oncol.

[8] Cumberbatch MGK (2018). Epidemiology of bladder cancer: a systematic review and contemporary update of risk factors in 2018. Eur Urol.

[9] Li R (2020). Macroscopic somatic clonal expansion in morphologically normal human urothelium. Science.

[10] Lawson ARJ (2020). Extensive heterogeneity in somatic mutation and selection in the human bladder. Science.

[11] Maraver A (2015). NOTCH pathway inactivation promotes bladder cancer progression. J Clin Invest.

[12] Chang SS (2016). Diagnosis and treatment of non-muscle invasive bladder cancer: AUA/SUO Guideline. J Urol.

[13] Kamat AM (2017). BCG-unresponsive non-muscle-invasive bladder cancer: recommendations from the IBCG. Nat Rev Urol.

[14] Lobo N (2021). 100 years of Bacillus Calmette-Guérin immunotherapy: from cattle to COVID-19. Nat Rev Urol.

[15] Daman AW (2025). Microbial cancer immunotherapy reprograms hematopoiesis to enhance myeloid-driven anti-tumor immunity. Cancer Cell.

[16] Antonelli AC (2020). Bacterial immunotherapy for cancer induces CD4-dependent tumor-specific immunity through tumor-intrinsic interferon-γ signaling. Proc Natl Acad Sci U S A.

[17] Strandgaard T (2022). Elevated T-cell exhaustion and urinary tumor DNA levels are associated with bacillus calmette-guérin failure in patients with non-muscle-invasive bladder cancer. Eur Urol.

[18] Duplisea JJ (2019). The development of interferon-based gene therapy for BCG unresponsive bladder cancer: from bench to bedside. World J Urol.

[19] Boorjian SA (2021). Intravesical nadofaragene firadenovec gene therapy for BCG-unresponsive non-muscle-invasive bladder cancer: a single-arm, open-label, repeat-dose clinical trial. Lancet Oncol.

[20] Ramesh N (2006). CG0070, a conditionally replicating granulocyte macrophage colony-stimulating factor--armed oncolytic adenovirus for the treatment of bladder cancer. Clin Cancer Res.

[21] Tyson MD (2025). Final results: bond-003 cohort C- phase 3, single-arm study of intravesical cretostimogene grenadenorepvec for high-risk bcg-unresponsive non-muscle invasive bladder cancer with carcinoma in situ. J Urol.

[22] Steinmetz AR (2025). Integrating gene therapy into the treatment paradigm for non-muscle invasive bladder cancer. Expert Opin Biol Ther.

[23] Steinberg G (2000). Efficacy and safety of valrubicin for the treatment of Bacillus Calmette-Guerin refractory carcinoma in situ of the bladder. The Valrubicin Study Group. J Urol.

[24] Daneshmand S (2025). Development of TAR-200: a novel targeted releasing system designed to provide sustained delivery of gemcitabine for patients with bladder cancer. Urol Oncol.

[25] Grimberg DC (2020). Overview of taris GemRIS, a novel drug delivery system for bladder cancer. Eur Urol Focus.

[26] Daneshmand S (2025). TAR-200 for bacillus calmette-guérin–unresponsive high-risk non–muscle-invasive bladder cancer: results from the phase IIb SunRISe-1 Study. J Clin Oncol.

[27] Vilaseca A (2023). LBA104 First safety and efficacy results of the TAR-210 erdafitinib (erda) intravesical delivery system in patients (pts) with non–muscle-invasive bladder cancer (NMIBC) with select FGFR alterations (alt). Ann Oncol.

[28] Loriot Y (2023). Erdafitinib or chemotherapy in advanced or metastatic urothelial carcinoma. N Engl J Med.

[29] Balar AV (2021). Pembrolizumab monotherapy for the treatment of high-risk non-muscle-invasive bladder cancer unresponsive to BCG (KEYNOTE-057): an open-label, single-arm, multicentre, phase 2 study. Lancet Oncol.

[30] Shore ND (2025). Sasanlimab plus BCG in BCG-naive, high-risk non-muscle invasive bladder cancer: the randomized phase 3 CREST trial. Nat Med.

[31] De Santis M (2025). Durvalumab in combination with BCG for BCG-naive, high-risk, non-muscle-invasive bladder cancer (POTOMAC): final analysis of a randomised, open-label, phase 3 trial. Lancet.

[32] Meghani K (2022). First-in-human intravesical delivery of pembrolizumab identifies immune activation in bladder cancer unresponsive to Bacillus Calmette-Guérin. Eur Urol.

[33] Folgosa Cooley L (2021). Survival by T stage for patients with localized bladder cancer: implications for future screening trials. Bladder Cancer.

[34] Advanced Bladder Cancer (ABC) Meta-Analysis Collaborators Group (2022). Adjuvant chemotherapy for muscle-invasive bladder cancer: a systematic review and meta-analysis of individual participant data from randomised controlled trials. Eur Urol.

[35] Sternberg CN (2015). Immediate versus deferred chemotherapy after radical cystectomy in patients with pT3-pT4 or N+ M0 urothelial carcinoma of the bladder (EORTC 30994): an intergroup, open-label, randomised phase 3 trial. Lancet Oncol.

[36] Grossman HB, Speights VO (2003). Neoadjuvant chemotherapy plus cystectomy compared with cystectomy alone for locally advanced bladder cancer. N Engl J Med.

[37] Bajorin DF (2021). Adjuvant nivolumab versus placebo in muscle-invasive urothelial carcinoma. N Engl J Med.

[38] Bellmunt J (2021). Adjuvant atezolizumab versus observation in muscle-invasive urothelial carcinoma (IMvigor010): a multicentre, open-label, randomised, phase 3 trial. Lancet Oncol.

[39] Powles T (2021). ctDNA guiding adjuvant immunotherapy in urothelial carcinoma. Nature.

[40] Powles T (2024). Pembrolizumab for advanced urothelial carcinoma: exploratory ctDNA biomarker analyses of the KEYNOTE-361 phase 3 trial. Nat Med.

[41] Powles T (2024). Perioperative durvalumab with neoadjuvant chemotherapy in operable bladder cancer. N Engl J Med.

[42] Powles T (2025). Circulating tumor DNA (ctDNA) in patients with muscle-invasive bladder cancer (MIBC) who received perioperative durvalumab (D) in NIAGARA. J Clin Oncol.

[43] Rosenberg J (2020). EV-101: a phase I study of single-agent enfortumab vedotin in patients with nectin-4-positive solid tumors, including metastatic urothelial carcinoma. J Clin Oncol.

[44] Hanna KS (2020). Clinical overview of enfortumab vedotin in the management of locally advanced or metastatic urothelial carcinoma. Drugs.

[45] Rosenberg JE (2019). Pivotal trial of enfortumab vedotin in urothelial carcinoma after platinum and anti-programmed death 1/programmed death ligand 1 therapy. J Clin Oncol.

[46] Powles T (2024). Enfortumab vedotin and pembrolizumab in untreated advanced urothelial cancer. N Engl J Med.

[47] Klümper N (2023). Membranous NECTIN-4 expression frequently decreases during metastatic spread of urothelial carcinoma and is associated with enfortumab vedotin resistance. Clin Cancer Res.

[48] Klümper N (2024). NECTIN4 amplification is frequent in solid tumors and predicts enfortumab vedotin response in metastatic urothelial cancer. J Clin Oncol.

[49] Vulsteke C (2025). LBA2 Perioperative (periop) enfortumab vedotin (EV) plus pembrolizumab (pembro) in participants (pts) with muscle-invasive bladder cancer (MIBC) who are cisplatin-ineligible: The phase III KEYNOTE-905 study. Ann Oncol.

[50] Raggi D (2026). HER2 and urothelial carcinoma: current understanding and future directions. Nat Rev Urol.

[51] Galsky MD (2024). 1967MO Preliminary efficacy and safety of disitamab vedotin (DV) with pembrolizumab (P) in treatment (Tx)-naive HER2-expressing, locally advanced or metastatic urothelial carcinoma (la/mUC): RC48G001 cohort C. Ann Oncol.

[52] Liu D (2023). Final analysis of multi-histology basket trial expansion of ado-trastuzumab emtansine in patients with HER2 amplified cancers. J Clin Oncol.

[53] Sheng X (2025). Disitamab vedotin plus toripalimab in HER2-expressing advanced urothelial cancer. N Engl J Med.

[54] Linscott JA (2024). The elusive horizon: biomarkers in urothelial carcinoma. Eur Urol.

[55] Rose KM (2023). Circulating and urinary tumour DNA in urothelial carcinoma - upper tract, lower tract and metastatic disease. Nat Rev Urol.

[56] Bicocca VT (2022). Urinary comprehensive genomic profiling: correlating urothelial carcinoma mutation with clinical risk and efficacy of intervention. J Clin Med.

[57] Salari K (2023). Development and multicenter case-control validation of urinary comprehensive genomic profiling for urothelial carcinoma diagnosis, surveillance, and risk-prediction. Clin Cancer Res.

[58] St-Laurent M-P (2025). Urine tumor DNA to stratify the risk of recurrence in patients treated with atezolizumab for bacillus calmette-guérin-unresponsive non-muscle-invasive bladder cancer. Eur Urol.

[59] Narayan VM (2025). Minimal residual disease detection with urine-derived DNA is prognostic for recurrence-free survival in bacillus calmette-guérin-unresponsive non-muscle-invasive bladder cancer treated with nadofaragene firadenovec. Eur Urol Oncol.

[60] Desai J (2024). Divarasib plus cetuximab in KRAS G12C-positive colorectal cancer: a phase 1b trial. Nat Med.

[61] Wang B (2025). Real-world experience with a commercial circulating tumor DNA assay in non-muscle-invasive bladder cancer. Eur Urol Oncol.

[62] Christensen E (2019). Early detection of metastatic relapse and monitoring of therapeutic efficacy by ultra-deep sequencing of plasma cell-free DNA in patients with urothelial bladder carcinoma. J Clin Oncol.

[63] Powles T (2024). Updated overall survival by circulating tumor DNA status from the phase 3 IMvigor010 trial: adjuvant atezolizumab versus observation in muscle-invasive urothelial carcinoma. Eur Urol.

[64] Jensen JB (2024). 1960O Identification of bladder cancer patients that could benefit from early post-cystectomy immunotherapy based on serial circulating tumour DNA (ctDNA) testing: preliminary results from the TOMBOLA trial. Ann Oncol.

[65] Powles T (2025). ctDNA-guided adjuvant atezolizumab in muscle-invasive bladder cancer. N Engl J Med.

[66] Huddart RA (2017). Clinical and patient-reported outcomes of SPARE - a randomised feasibility study of selective bladder preservation versus radical cystectomy. BJU Int.

[67] Kougioumtzopoulou A (2024). 1961O Nivolumab plus chemoradiotherapy in patients with non-metastatic muscle-invasive bladder cancer (nmMIBC), not undergoing cystectomy: a phase II, randomized study by the Hellenic GU Cancer Group. Ann Oncol.

[68] Balar AV (2021). Pembrolizumab (pembro) in combination with gemcitabine (Gem) and concurrent hypofractionated radiation therapy (RT) as bladder sparing treatment for muscle-invasive urothelial cancer of the bladder (MIBC): a multicenter phase 2 trial. J Clin Oncol.

[69] Gupta S (2024). Phase 3 KEYNOTE-992 study of pembrolizumab plus chemoradiotherapy versus placebo plus chemoradiotherapy in patients with muscle-invasive bladder cancer (MIBC). J Clin Oncol.

[70] Iyer G (2022). A phase II study of gemcitabine plus cisplatin chemotherapy in patients with muscle-invasive bladder cancer with bladder preservation for those patients whose tumors harbor deleterious DNA damage response (DDR) gene alterations (Alliance A031701). J Clin Oncol.

[71] Geynisman DM (2025). Phase II trial of risk-enabled therapy after neoadjuvant chemotherapy for muscle-invasive bladder cancer (RETAIN 1). J Clin Oncol.

[72] Ghatalia P (2025). A phase 2 trial of risk enabled therapy after neoadjuvant chemo-immunotherapy for muscle-invasive bladder cancer (RETAIN-2). J Clin Oncol.

[73] Galsky MD (2023). Gemcitabine and cisplatin plus nivolumab as organ-sparing treatment for muscle-invasive bladder cancer: a phase 2 trial. Nat Med.

